# Supplementary material for: Software Assisted Multi-Tiered Mass Spectrometry Identification of Compounds in Traditional Chinese Medicine: Dalbergia odorifera as an Example
Source: Molecules. 2022 Apr 4;27(7):2333. doi: 10.3390/molecules27072333 (PMC9000885; doi:10.3390/molecules27072333)
Supplement: Supplementary file 1 [file molecules-27-02333-s001.zip › molecules-1654811-supplementary.pdf]

Supporting information

Software assisted multi-tiered mass spectrometry identification of compounds in Traditional Chinese Medicine: Dalbergia odorifera as an example

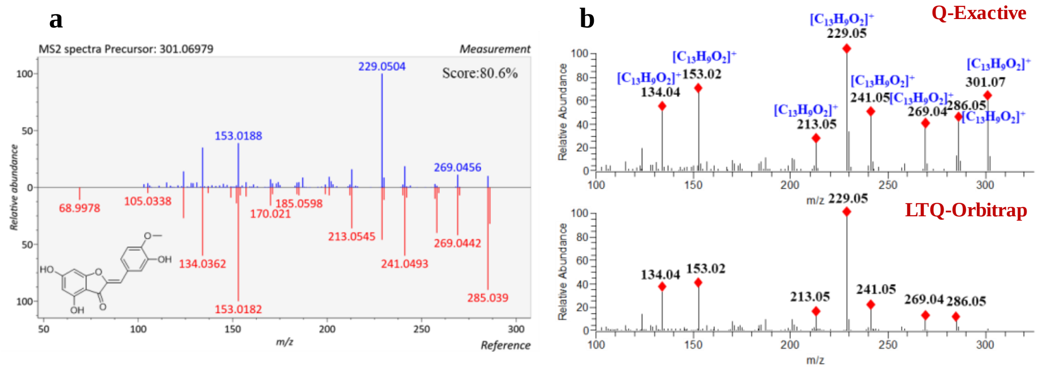

Figure S1. Fragment spectra of compound **89**. (**a**) Matching diagram of compound **89** in MS-DIAL; (**b**) Fragment spectra of compound **89** in different instruments.

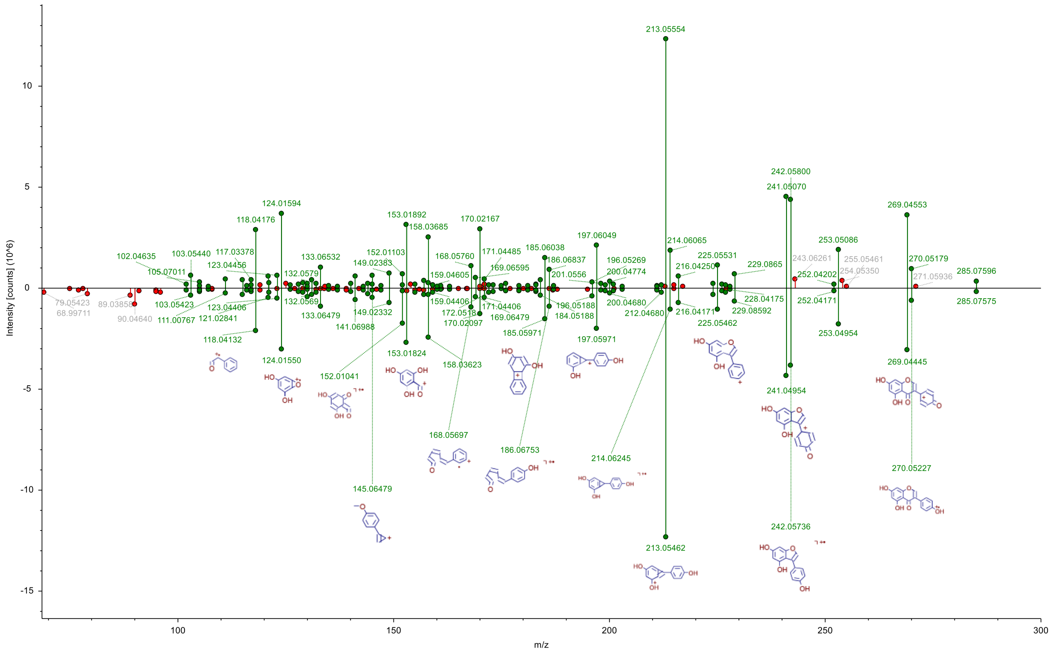

Figure S2. Matching diagram of compound **147** in Compound Discoverer 3.3.

Structure	Name	Formula	Molecular Weight	ΔMass [Da]	ΔMass [ppm]	CSID	# References	mzLogic Score
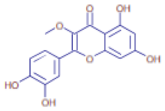	324509	C16 H12 O7	316.05830	-0.00027	-0.87	4444273	193	78.1
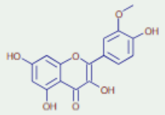	Isorhamnetin	C16 H12 O7	316.05830	-0.00027	-0.87	4444973	516	77.7
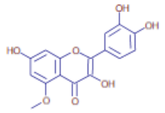	Azaleatin	C16 H12 O7	316.05830	-0.00027	-0.87	4444923	60	77.1
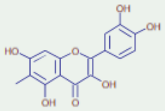	Pinoquercetin	C16 H12 O7	316.05830	-0.00027	-0.87	4444998	30	77.1
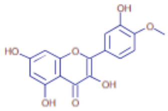	TAMARIXETIN	C16 H12 O7	316.05830	-0.00027	-0.87	4445016	109	77.1

Figure S3. The top 5 candidates of compound **27**.

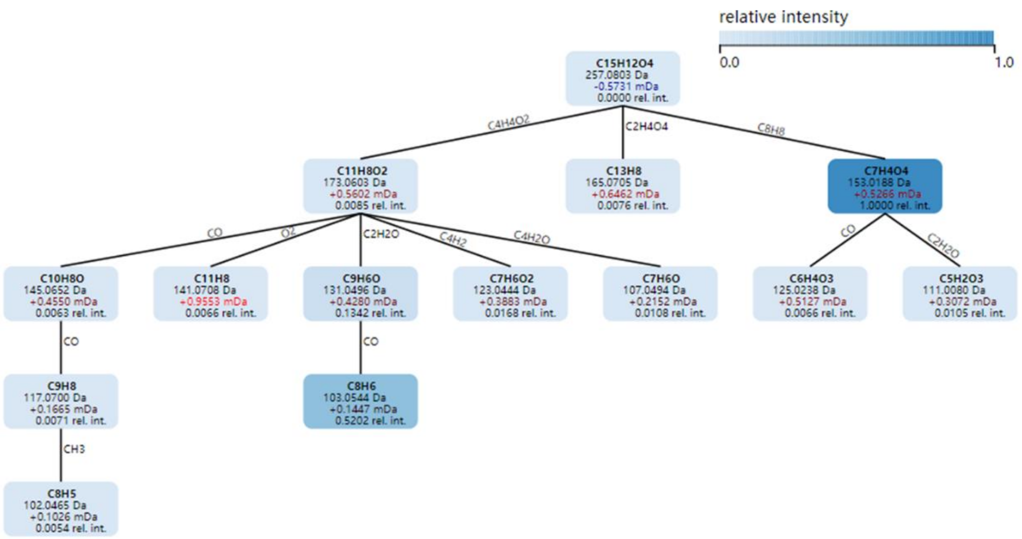

Figure S4. The fragment tree of compound **138**.

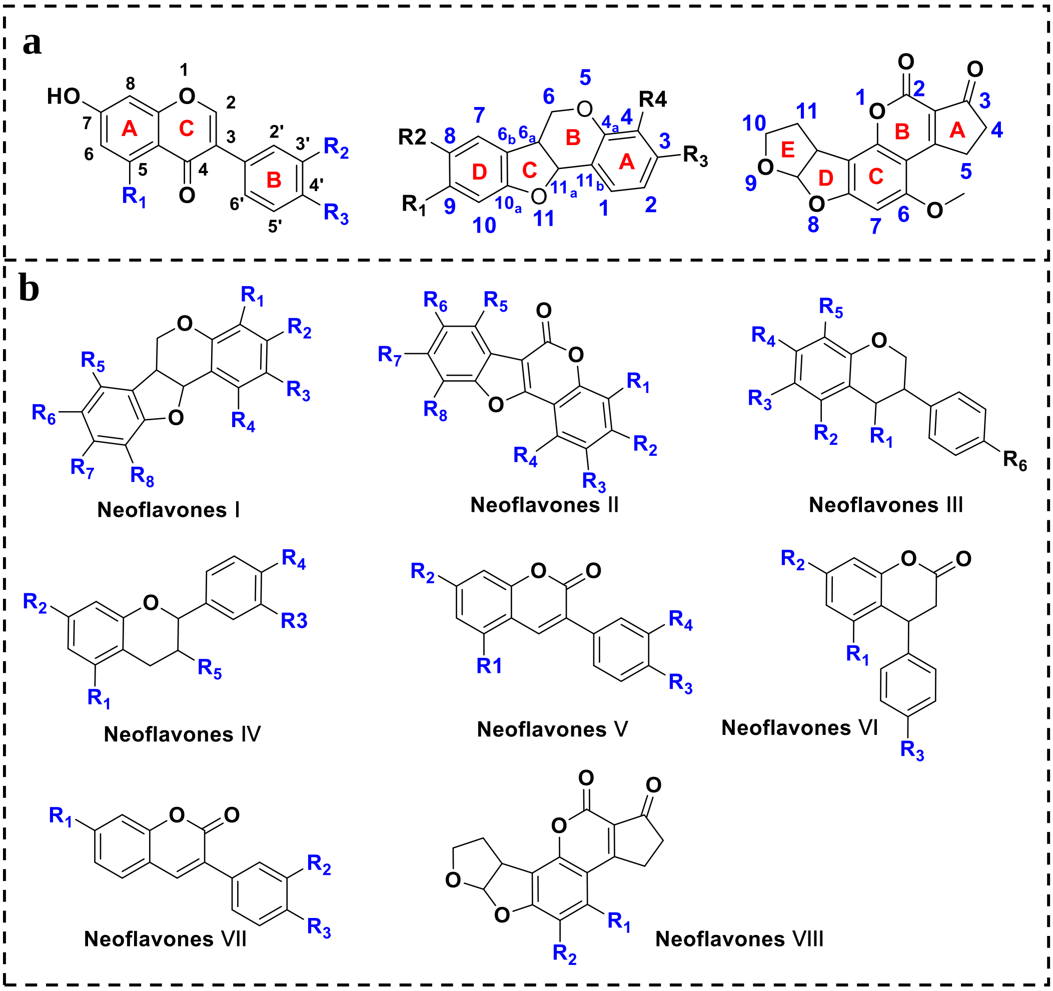

Figure S5. Structure and structure numbering of different subtypes of flavonoids. (**a**) Structural numbers of different subtypes of flavonoids; (**b**)

Structures of eight types of neoflavonoids.

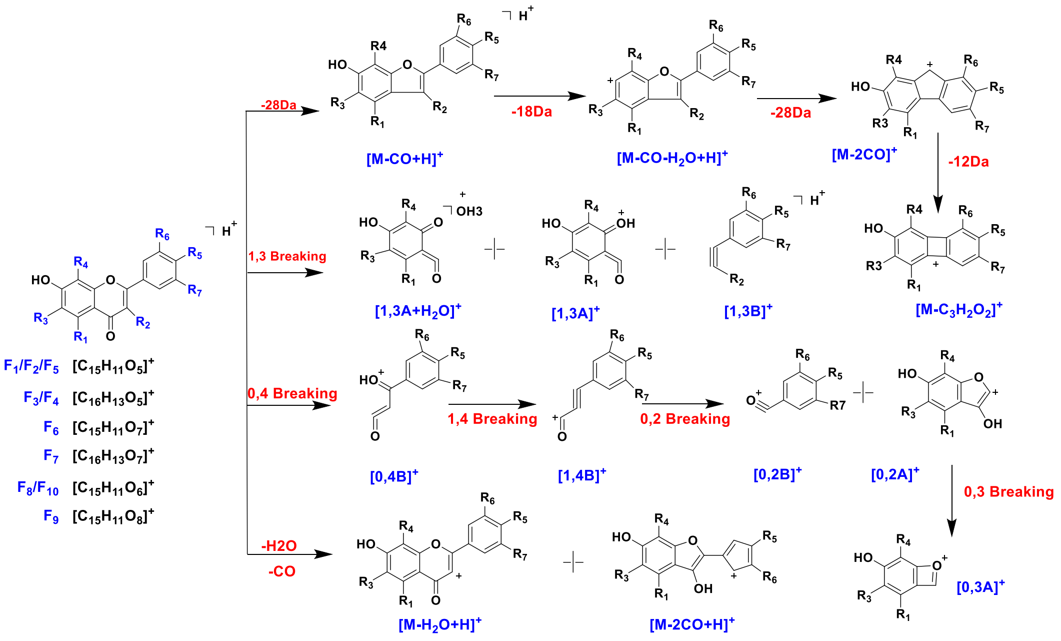

Figure S6. The proposed MS fragmentation pathways of compounds F1-F10.

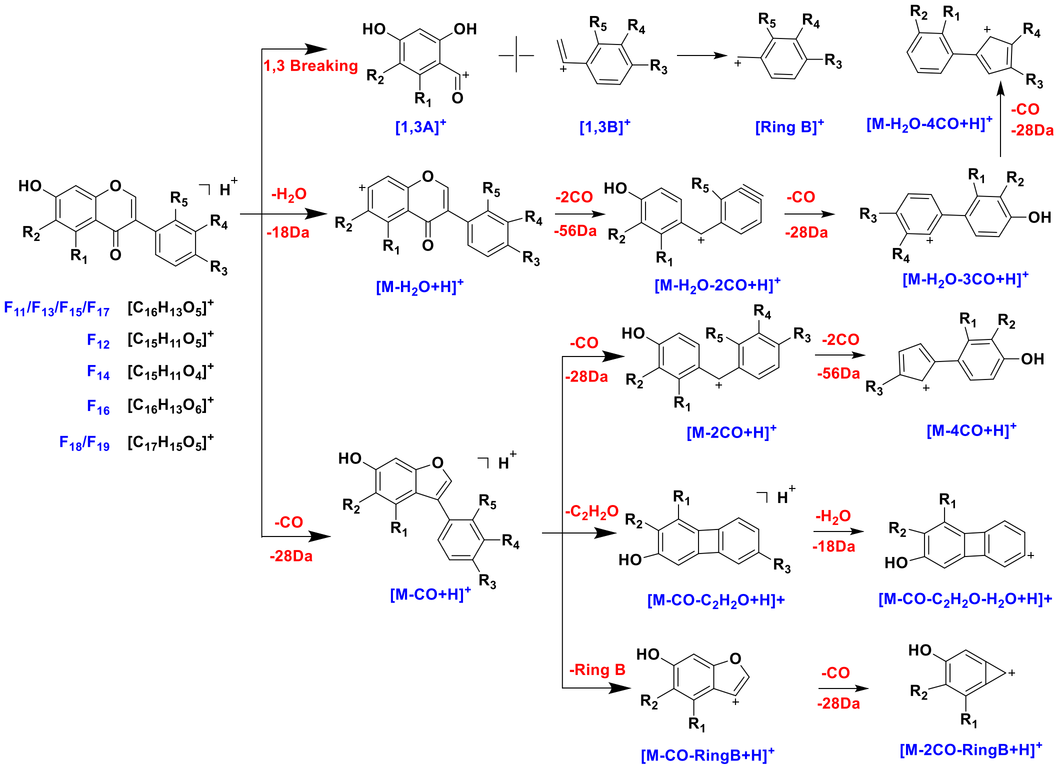

Figure S7. The proposed MS fragmentation pathways of compounds F11-F19.

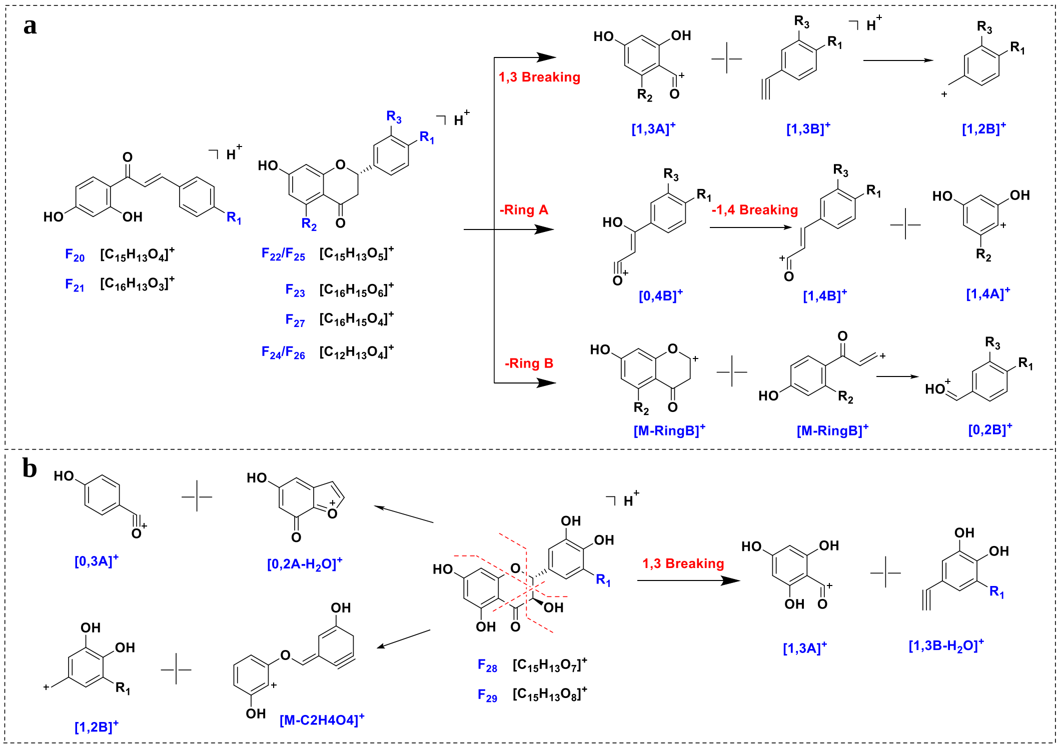

Figure S8. The proposed MS fragmentation pathways of compounds F_{20}-F_{29}. (**a**) F_{20}-F_{27}; (**b**) F_{28}-F_{29}.
